# Biocompatibility and Inflammatory Potential of Titanium Alloys Cultivated with Human Osteoblasts, Fibroblasts and Macrophages

**DOI:** 10.3390/ma10010052

**Published:** 2017-01-10

**Authors:** Jana Markhoff, Martin Krogull, Christian Schulze, Christian Rotsch, Sandra Hunger, Rainer Bader

**Affiliations:** 1 Biomechanics and Implant Technology Laboratory, Department of Orthopaedics, University Medicine Rostock, Doberaner Strasse 142, 18057 Rostock, Germany; martin.krogull2@uni-rostock.de (M.K.); christian_schulze@med.uni-rostock.de (C.S.); rainer.bader@med.uni-rostock.de (R.B.); 2 Department Medical Engineering, Fraunhofer Institute for Machine Tools and Forming Technology IWU, Nöthnitzer Strasse 44, 01187 Dresden, Germany; christian.rotsch@iwu.fraunhofer.de (C.R.); sandra.hunger@iwu.fraunhofer.de (S.H.)

**Keywords:** NiTi, DLC, Ti6Al4V, cell viability, human osteoblasts, macrophages

## Abstract

The biomaterials used to maintain or replace functions in the human body consist mainly of metals, ceramics or polymers. In orthopedic surgery, metallic materials, especially titanium and its alloys, are the most common, due to their excellent mechanical properties, corrosion resistance, and biocompatibility. Aside from the established Ti6Al4V alloy, shape memory materials such as nickel-titanium (NiTi) have risen in importance, but are also discussed because of the adverse effects of nickel ions. These might be reduced by specific surface modifications. In the present in vitro study, the osteoblastic cell line MG-63 as well as primary human osteoblasts, fibroblasts, and macrophages were cultured on titanium alloys (forged Ti6Al4V, additive manufactured Ti6Al4V, NiTi, and Diamond-Like-Carbon (DLC)-coated NiTi) to verify their specific biocompatibility and inflammatory potential. Additive manufactured Ti6Al4V and NiTi revealed the highest levels of metabolic cell activity. DLC-coated NiTi appeared as a suitable surface for cell growth, showing the highest collagen production. None of the implant materials caused a strong inflammatory response. In general, no distinct cell-specific response could be observed for the materials and surface coating used. In summary, all tested titanium alloys seem to be biologically appropriate for application in orthopedic surgery.

## 1. Introduction

Biomaterials are used to maintain or replace a wide range of functions in the human body that have been lost due to tumors, fractures, infections, or aging [1,2,3]. In orthopedic surgery, the materials used consist mainly of metals, ceramics, or polymers [4]. Thereby, metallic materials can be made of stainless steel, titanium or alloys of titanium, cobalt–chromium, magnesium, tantalum, or niobium, for example [5]. Titanium and its alloys are the most common materials, comprising 70%–80% of all used materials [6,7,8]. Due to their excellent mechanical properties, corrosion resistance, and biocompatibility, these materials are used in load-bearing areas such as orthodontics, orthopedics, and gastroenterology as well as for cardiovascular and reconstructive purposes [5,6,9,10,11,12]. Furthermore, components made of titanium can be easily manufactured in various shapes and textures [6]. Commercial pure titanium and Ti6Al4V were established in the 1950s and offer excellent physical and chemical properties; then, shape memory alloys like nickel-titanium (as nitinol with a nickel content of 49%–51%) were first reported in the late 1960s [13] and have risen in importance in the last decades [14]. This is related to their favorable properties like the shape memory effect (SME), good biocompatibility, and superelasticity of up to 8% [15], with mechanical values (elastic modulus and compressive strength) close to those of bone tissue [16,17,18] as well as beneficial corrosion and wear resistance [15]. Nevertheless, fabrication of NiTi is difficult and expensive [5,15]. NiTi alloys are also discussed, because of the possible toxic, allergenic, and carcinogenic properties of nickel ions [5,16,19]. To create an ion diffusion barrier, several surface modifications have been taken into account [14,16], for example chemical, electrochemical, or heat treatment as well as irradiation or coatings like diamond-like carbon (DLC) [14,20,21,22]. DLC coatings are reported to be chemically inert with good biocompatibility [20]. For initial cell attachment and growth, the surface properties (roughness, surface chemical structure, and wettability) play an important role [6,14], since they even result in a cell-specific response [10,23]. It is known that osteoblasts prefer rougher surfaces while fibroblast growth is increased on smoother ones, for example [10]. Since human tissue comprises specific cell types, cells of different origins and with various functions should be considered for preclinical testing of specific biomaterials. Primary human cells may be preferred since cell lines may not fully display the physiological situation [24]. Osteoblasts are cells of the lineage of mesenchymal stem cells and are responsible for the formation of new bone [25]. Fibroblasts and mesenchymal stromal cells are involved in inflammatory processes occurring in all types of tissues [26]. Macrophages play a dominant role in inflammation and interaction with implanted materials and represent an important type of cell for biocompatibility testing [27]. Macrophages develop from monocytic cells, which are a multifunctional fraction of peripheral blood mononuclear cells (PBMCs) [28].

In the present in vitro study, the osteoblastic cell line MG-63 as well as human osteoblasts, human fibroblasts, and human macrophages were cultured on four titanium alloys (forged Ti6Al4V, additive manufactured Ti6Al4V, NiTi, and DLC-coated NiTi) to verify their specific biocompatibility and inflammatory potential.

## 2. Materials and Methods

### 2.1. Test Samples

The forged Ti6Al4V samples were provided by DOT GmbH, Rostock, Germany. The selective laser melted (SLM)-manufactured Ti6Al4V pellets were manufactured with an EOS M 280 and provided by Proto Labs Eschenlohe GmbH, Eschenlohe, Germany. The samples were heat-treated after the manufacturing process (protective gas atmosphere argon, slow cooling in oven under inert gas) and run through a multi-level post-processing (removing the support-structures, grinding of supported surfaces, sandblasting of the samples with 20–45 μm grain size), cleaning (removing of loose particles) and sterilization process. We used NiTi-samples (Memry GmbH, Weil, Germany), which are biocompatible commercially available materials. NiTi-samples were oxide-free etched by the manufacturer. The DLC coating of the conventionally available biocompatible NiTi (Type “S” by Memry GmbH) with a nickel content of about 50% was provided by EC EUROP COATING GmbH (Hohenlockstedt, Germany). The coating was realized by a physical vapor deposition (PVD) process (dc magnetron sputtering; coating temperature: 180–200 °C; coating thickness: about 2–4 µm).

All test samples exhibited similar geometries and were cleaned in an ultrasonic bath and sterilized via gamma sterilization with 25 kGy. Surface roughness was measured using a profilometer (Hommel–Etamic T1000, Jenoptik AG, Jena, Germany). The parameters “mean roughness index” (Ra) and “average surface roughness” (Rz) were determined (Table 1).

**Figure 1:**
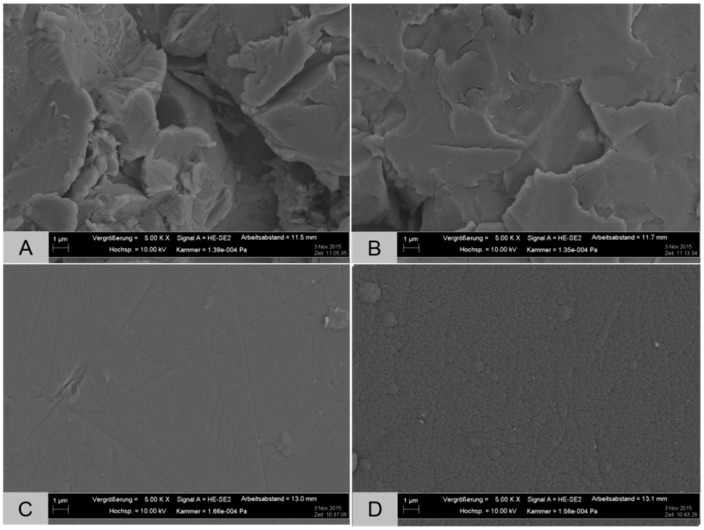
FESEM images of the test pellets: (**A**) Ti6Al4V; (**B**) Ti6Al4V SLM; (**C**) NiTi; and (**D**) NiTi + DLC (magnification: 5.00 kX, bar: 1 µm).

Field emission scanning electron microscopy (FESEM) images of the sample surfaces were generated using a sputter coater (Leica SCD004, Wetzlar, Germany) and Merlin VP compact microscope (Carl Zeiss AG, Oberkochen, Germany) (Figure 1).

### 2.2. Cell Isolation

For the in vitro tests, a cell line and three human types of cells were used. The use of all human cell types was approved by the Local Ethical Committee of the University Medicine Rostock (registration numbers: for osteoblasts: A 2010-10; for fibroblasts: A 2013-0092; and for macrophages: A 2011-140). Characteristics of the human cell donors are mentioned in Table 2. Cell cultivation was carried out in an incubator (Binder GmbH, Tuttlingen, Germany) under simulated in vivo conditions at 5% CO_2_, 21% O_2_, and 37 °C with regular medium changes unless otherwise stated. Tissue culture polystyrene (TCPS) served as a common surface for growth control. 

The bone cell line MG-63 was ordered from ATCC (American Type Culture Collection, Manassas, VA, USA). MG-63 is an established cell line, isolated from the osteosarcoma of a 14-year old Caucasian male patient. MG-63 cells were cultured in Dulbecco’s Modified Eagle’s Medium (DMEM) with 10% fetal calf serum (FCS), 1% penicillin/streptomycin, 1% amphotericin B, 1% HEPES buffer (2-(4-(2-hydroxyethyl)-1-piperazineethanesulfonic acid) (all: Gibco-Invitrogen, Darmstadt, Germany). The test samples were directly seeded with MG-63 cells at a density of 20,000 cells per 48 wells in 500 µL of culture medium in two wells per donor for every configuration.

**Figure 2:**
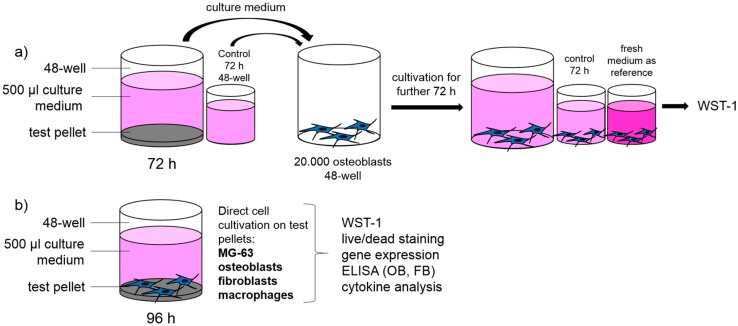
Schematic experiment set-up: (**a**) verification of potential cytopathic substances; and (**b**) analysis of biocompatibility of different titanium alloys with four types of cells.

The isolation of human primary osteoblasts was performed as previously described by Lochner et al. [29]. After patient agreement, the femoral heads of the patients undergoing primary total hip replacement in our in-house operating theatre were collected and cells were isolated from the spongiosa. Cultivation was done in osteogenic cell culture medium (Minimum Essential Medium (MEM) Dulbecco, Biochrom AG, Berlin, Germany) with 10% FCS, 1% penicillin/streptomycin, 1% amphotericin B, and 1% HEPES buffer (all from Gibco-Invitrogen, Darmstadt, Germany), including osteogenic additives (dexamethasone (100 nM), l-ascorbic acid (50 µg/mL), and β-glycerophosphate (10 mM) (all from Sigma-Aldrich, Munich, Germany)). To verify the osteogenic character of the isolated cells, alkaline phosphatase staining with fuchsin substrate chromogen (DAKO, Hamburg, Germany) was carried out. The test samples were directly seeded with osteoblasts at a density of 20,000 cells (third passage) per 48 wells in 500 µL of culture medium with two wells per donor for every configuration (Figure 2b). Furthermore, to verify whether cytopathic substances are emitted into the culture medium, all test samples were incubated in culture medium during 72 h with an additional medium control without pellet. Afterwards, the supernatants were transferred onto human osteoblasts (20,000 cells per 48 wells in 500 µL) for a further 72 h of cultivation in two wells per donor for every configuration (Figure 2a).

Human fibroblasts were isolated from skin biopsies (breast, eyelid) provided by a local clinic for aesthetic surgeries. Redundant adipose tissue was removed and the remaining tissue was cut into equal segments (edge length: 2–3 mm). Then, skin pieces were transferred to six-well plates (2–3 bits per well) with the epidermis upwards. After 20 min of surface drying, skin was overlaid with 3 mL of DMEM medium (with Glutamax, 10% FCS, 1% penicillin/streptomycin, and 1% amphotericin B (all from Gibco-Invitrogen, Darmstadt, Germany)). After three weeks, cells were transferred to tissue culture flasks and cryo-preserved after further confluence. The test pellets were directly seeded with fibroblasts at a density of 20,000 cells (fifth passage) per 48 wells in 500 µL of culture medium in two wells per donor for every configuration.

Furthermore, human buffy coats from blood donations were provided by the Institute of Transfusion Medicine, University Medicine Rostock and used after patient agreement for isolation of PBMCs. The buffy coats were separated in different directions by means of density gradient centrifugation (Ficoll Hypaque method) by lymphocyte separation medium (Histopaque-1077, Sigma-Aldrich, Hamburg, Germany). The interphase containing the PBMC-like lymphocytes and monocytes (density: 1.07 g/mL) was extracted by means of a Pasteur pipette. After two washing steps in phosphate-buffered saline (PBS, Biochrom GmbH, Berlin, Germany), the cells were seeded in monolayers (1 × 10^7^ cells per well in 3 mL) in six-well suspension culture plates (Cellstar cell-repellent surface, Greiner Bio-One, Frickenhausen, Germany) using Roswell Park Memorial Institute (RPMI) medium 1640 (Biochrom, Berlin, Germany) containing 10% FCS, 1% penicillin/streptomycin (all from Gibco-Invitrogen, Darmstadt, Germany), and 2% l-glutamine (PAA Laboratories GmbH, Coelbe, Germany). After seven days of cultivation, suspension cells were transferred to a standard 48-well culture plate and directly cultured on test pellets with a density of 4 × 10^5^ cells per well in 500 µL of (MEM Alpha-Medium (1×), Life Technologies GmbH, Darmstadt, Germany) with 10% FCS and 2% penicillin/streptomycin in two wells per donor for every configuration.

### 2.3. Cell Biological Testing

#### 2.3.1. Metabolic Activity and Live/Dead Staining

The metabolic activity of cells was determined via mitochondrial dehydrogenase activity (WST-1 test) (Roche, Grenzach–Wyhlen, Germany) after 96 h. The tetrazolium salt WST (water soluble tetrazolium) is transformed into formazan by mitochondrial succinate dehydrogenase from metabolically active cells. The adsorption, which was directly proportional to the metabolic cell activity, was measured at 450 nm in a Tecan reader (Infinite F200 Pro, Männedorf, Switzerland). Qualitative cell viability was analyzed by live/dead staining with two fluorescent dyes. Calcein-Acetyoxymethyl (AM) visualizes vital cells and ethidium homodimer-1 visualizes dead ones (Live/Dead Cell Viability Assay, Invitrogen, Darmstadt, Germany). For imaging, an inverted microscope (Nikon TS 100, Nikon GmbH, Duesseldorf, Germany) was used.

#### 2.3.2. Gene Expression Analysis

Human osteoclasts, fibroblasts, and macrophages were isolated from the test pellets to obtain the total RNA via TRIzol reagent (Invitrogen, Darmstadt, Germany) and the Direct-zol RNA kit (Zymo Research, Freiburg, Germany), according to the manufacturer’s instructions, with optional DNAse digestion. RNA concentrations were determined using Tecan’s NanoQuant plate and the Tecan reader (Infinite F200 Pro, Männedorf, Switzerland). Afterwards, 250 ng of total RNA was retrotranscribed to cDNA using the High Capacity cDNA Reverse Transcription Kit (Applied Biosystems, Darmstadt, Germany) with 10× RT buffer, 25× dNTP Mix (100 mM), RT random primers, MultiScribe^®^ Reverse Transcriptase (50 U/µL), and Diethylpyrocarbonate (DEPC) water in a final volume of 20 μL. This suspension was incubated for 10 min at 25 °C, 120 min at 37 °C, and finally 15 s at 85 °C in a thermocycler (Personal Thermocycler, Biometra, Göttingen, Germany). One microliter of cDNA was used as the template for polymerase chain reaction (PCR) in triple. These reactions were performed with DEPC water, 0.5 µL of each forward and reverse primer (Sigma-Aldrich, Hamburg, Germany), and innuMIX qPCR MasterMix SyGreen (Analytik Jena, Jena, Germany) including high specific Taq DNA polymerase, high quality dNTPs, and intercalating dyestuff, in a final reaction volume of 20 μL. The primer details are summarized in Table 3. The quantitative PCR reaction was performed at 95 °C for 3 min, 95 °C for 5 s, and 60 °C for 30 s over 40 cycles via qTower 2.0 (Analytik Jena, Jena, Germany). β-actin (osteoblasts and fibroblasts) and Hypoxanthine-guanine phosphoribosyltransferase (HPRT) (macrophages) were chosen as housekeeping genes. 

#### 2.3.3. Enzyme-Linked Immunosorbent Assays

Enzyme-linked immunosorbent assays (ELISAs) were used to verify protein syntheses of pro-collagen type I (Metra C1CP EIA Kit, Quidel, Buende, Germany) and MMP-1 (Human MMP-1 ELISA, RayBiotech, Köln, Germany) in human osteoblasts and fibroblasts. Protein content in the culture supernatants was determined according to the manufacturer’s instructions after 96 h of cultivation.

#### 2.3.4. Cytokine Analysis

A cytokine multiplex assay (IL-6, IL-8, MCP-1, TNF-α) (Bio-Rad, Munich, Germany) was done according to the manufacturer’s instructions to verify a possible immune-stimulatory effect of the test pellets. In brief, multi-cytokine detection in the culture supernatants was conducted by means of microsphere beads interlinking several cytokines via specific monoclonal antibodies and fluorescent dyes. The appropriate cytokine concentration was measured using the BioPlex 200 System (Bio-Rad, Munich, Germany) and calculated based on a recombinant standard curve of the assay. The reader combines two lasers (reporter laser 532 nm, classification laser 635 nm) and high-throughput fluidics (<100 µL/s) to distinguish several color-coded beads. 

#### 2.3.5. Statistical Analysis

The statistical significance was calculated using IBM^®^ SPSS^®^ Statistics Version 20 (IBM Corp., New York, NY, USA). Multiple comparison procedures were carried out by the ANOVA Post Hoc LSD test. Data are shown as box plots. Boxes denote interquartile ranges, horizontal lines within the boxes denote medians, and whiskers denote minimum and maximum values. Values of *p* < 0.05 were set to be significant.

## 3. Results

**Figure 3:**
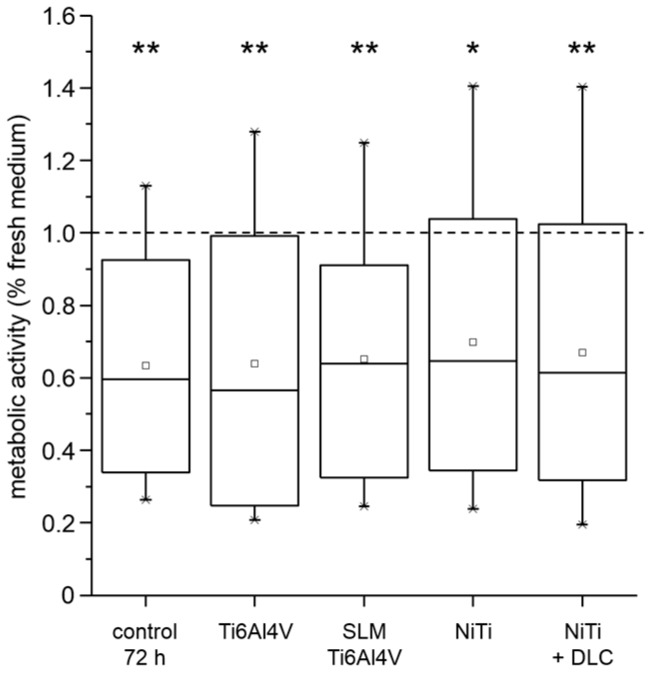
Metabolic activity of human osteoblasts (*n* = 4) cultured in pre-incubated (72 h) cell culture medium and incubated supernatants (72 h) of different test pellets (forged Ti6Al4V, Ti6Al4V SLM, NiTi, and NiTi + DLC) compared to fresh medium as reference (dotted line). Boxes denote interquartile ranges, horizontal lines within the boxes denote medians, and whiskers denote minimum and maximum values. For statistical analysis, ANOVA was conducted. Data were compared to the fresh medium control (100%, * *p*). * *p* < 0.05, ** *p* < 0.01.

Various cell types (MG-63; human osteoblasts, fibroblasts, and macrophages) were used to verify the biocompatibility of several metallic test samples (forged Ti6Al4V, Ti6Al4V SLM, NiTi, and NiTi + DLC coating). After cultivation of human osteoblasts for 72 h in pre-incubated sample medium, all groups including the 72 h medium control showed a significant decrease of metabolic activity of nearly 40% compared to the fresh medium reference (Figure 3).

**Figure 4:**
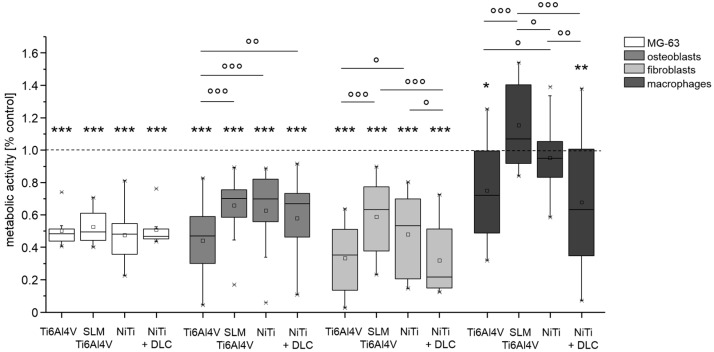
Metabolic activity of MG63 cells (*n* = 8), human osteoblasts (*n* = 8), human fibroblasts (*n* = 4), and human macrophages (*n* = 4) after 96 h of cultivation on several test pellets (forged Ti6Al4V, Ti6Al4V SLM, NiTi, NiTi + DLC). Boxes denote interquartile ranges, horizontal lines within the boxes denote medians, and whiskers denote minimum and maximum values. For statistical analysis, ANOVA was conducted. Data were compared to the respective growth control (100%, * *p*) and against each other (° *p*). * *p* < 0.05, ** *p* < 0.01, *** *p* ≤ 0.001; ° *p* < 0.05, °° *p* < 0.01, °°° *p* ≤ 0.001.

**Figure 5:**
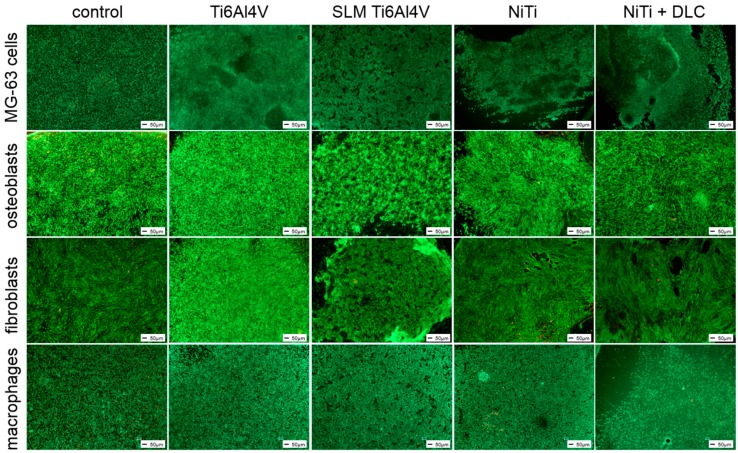
Live/dead staining of MG-63 cells, human osteoblasts, fibroblasts, and macrophages on different test pellets (forged Ti6Al4V, Ti6Al4V SLM, NiTi, and NiTi + DLC). Living cells are displayed in green, dead ones in red. Scale bar: 50 µm.

All cell types were seeded directly on the test samples using TCPS as a common surface for growth control. Except for macrophages, the metabolic activity of cells cultured on the metallic test samples was significantly lower than the growth control. Cultivation of cell line MG-63 on the test samples revealed no significant differences between the test pellets, but compared to the growth control, metabolic activity was significantly decreased by half for all groups (Figure 4, white bars). In general, the human cells showed similar results regarding differences between materials. The forged Ti6Al4V and NiTi + DLC coating resulted in significantly lower activities compared to Ti6Al4V SLM and NiTi, respectively (Figure 4, all gray bars). Metabolic activity on Ti6Al4V SLM and NiTi was similar for human osteoblasts (66% and 63%) and highest on Ti6Al4V SLM with human fibroblasts (59%) and especially human macrophages (115%). These results are partly confirmed by live/dead staining (Figure 5).

**Figure 6:**
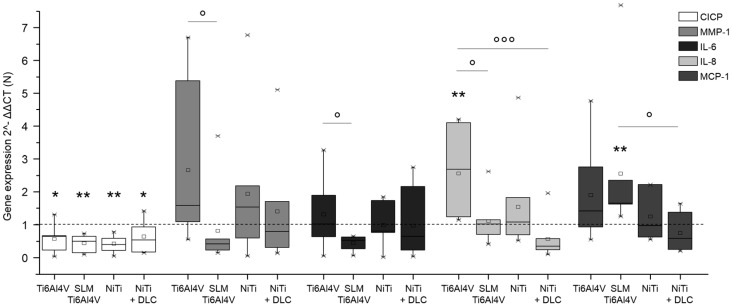
Gene expression (CICP, MMP-1, IL-6, IL-8, and MCP-1) of human osteoblasts (*n* = 8) after 96 h of cultivation on several test pellets (forged Ti6Al4V, Ti6Al4V SLM, NiTi, and NiTi + DLC). Boxes denote interquartile ranges, horizontal lines within the boxes denote medians, and whiskers denote minimum and maximum values. Data are normalized to the growth control (=100%, dotted line). For statistical analysis, ANOVA was conducted. A *p*-value < 0.05 was considered statistically significant. Comparison to respective growth control: * *p* < 0.05, ** *p* < 0.01. Comparison between several test pellets: ° *p* < 0.05, °°° *p* ≤ 0.001.

**Figure 7:**
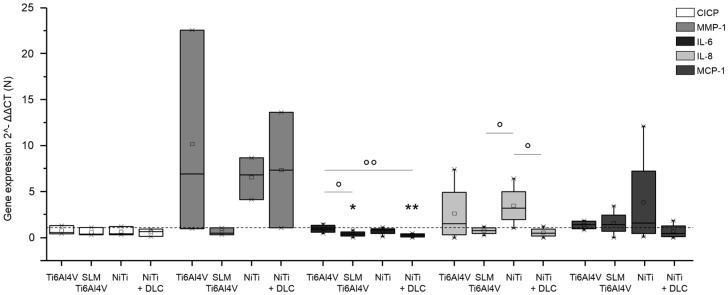
Gene expression (CICP, MMP-1, IL-6, IL-8, and MCP-1) of human fibroblasts (*n* ≥ 3) after 96 h of cultivation on several test pellets (forged Ti6Al4V, Ti6Al4V SLM, NiTi, and NiTi + DLC). Boxes denote interquartile ranges, horizontal lines within the boxes denote medians, and whiskers denote minimum and maximum values. Data are normalized to the growth control (=100%, dotted line). For statistical analysis, ANOVA was conducted. A *p*-value < 0.05 was considered statistically significant. Comparison to growth control: * *p* < 0.05, ** *p* < 0.01. Comparison between several test pellets: ° *p* < 0.05, °° *p* < 0.01.

Gene expression analysis was conducted for pro-collagen type I as the main component of bone matrix, MMP-1 as its degrading enzyme, and several cytokines (IL-6, IL-8, MCP-1, and TNF-α) to verify inflammatory cytokine secretion. Both human osteoblasts and fibroblasts presented a decreased expression of pro-collagen type I for all groups in similar to TCPS and without significant differences between the several test samples (Figure 6 and Figure 7, both white bars). MMP-1 expression was increased on forged Ti6Al4V and decreased on SLM manufactured Ti6Al4V for both types of cells compared to the control. Expression of MMP-1 on NiTi surfaces with and without DLC coating was slightly increased in osteoblasts and obviously higher compared to control in fibroblasts, but without distinct differences. Cytokine expression was similar for those cells, as IL-6 and IL-8 were increased with forged Ti6Al4V and NiTi as well as decreased with SLM Ti6Al4V and NiTi + DLC, and thus varied among the test samples. Furthermore, expression of MCP-1 was slightly higher on the Ti6Al4V surfaces compared to the control and similar or lower on NiTi surfaces. 

**Figure 8:**
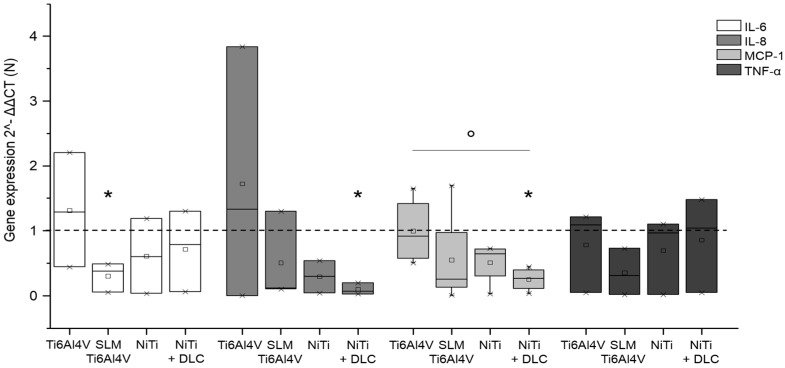
Gene expression (IL-6, IL-8, MCP-1, and TNF-α) of human macrophages (*n* ≥ 3) after 96 h of cultivation on several test pellets (forged Ti6Al4V, Ti6Al4V SLM, NiTi, and NiTi + DLC). Boxes denote interquartile ranges, horizontal lines within the boxes denote medians, and whiskers denote minimum and maximum values. Data are normalized to the growth control (=100%, dotted line). For statistical analysis, ANOVA was conducted. A *p*-value < 0.05 was considered statistically significant. Comparison to growth control: * *p* < 0.05. Comparison between several test pellets: ° *p* < 0.05.

Gene expression analysis for macrophages on metallic test samples revealed similar or significantly decreased expression compared to the control for all proved cytokines (Figure 8). In general, the highest expression of all cytokines was proven with forged Ti6Al4V. Macrophages on SLM Ti6Al4V expressed the lowest levels of IL-6 and TNF-α and expression of IL-8 and MCP-1 was the lowest with NiTi.

**Figure 9:**
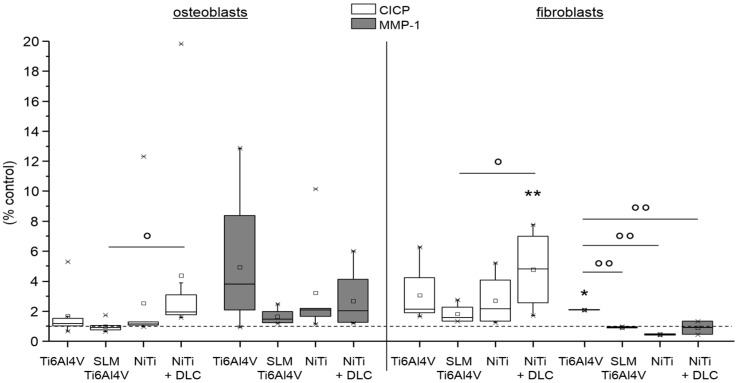
Pro-collagen type I synthesis (white) and MMP-1 synthesis (gray) of human osteoblasts (*n* = 8, left) and human fibroblasts (*n* ≥ 2, right) after 96 h of cultivation on several test pellets (forged Ti6Al4V, Ti6Al4V SLM, NiTi, NiTi + DLC). Boxes denote interquartile ranges, horizontal lines within the boxes denote medians, and whiskers denote minimum and maximum values. For statistical analysis, ANOVA was conducted. Data were compared with the respective growth control (100%, * *p*) and with each other (° *p*). * *p* < 0.05, ** *p* < 0.01; ° *p* < 0.05, °° *p* < 0.01.

For osteoblasts and fibroblasts, protein synthesis of pro-collagen type I and MMP-1 was determined. Collagen synthesis of human osteoblasts was nearly unaffected on Ti6Al4V surfaces, but was increased on NiTi ones compared to TCPS (Figure 9, left, white bars). In contrast, MMP-1 synthesis was increased in all groups, being highest on forged Ti6Al4V and similar in the other groups (Figure 9, left, gray bars). The pro-collagen type I synthesis of human fibroblasts was higher than on TCPS, with synthesis of all test samples being significantly increased on NiTi + DLC (Figure 9, right, white bars). MMP-1 synthesis was increased on NiTi surfaces compared to TCPS, and was highest on NiTi + DLC (Figure 9, right, gray bars).

**Figure 10:**
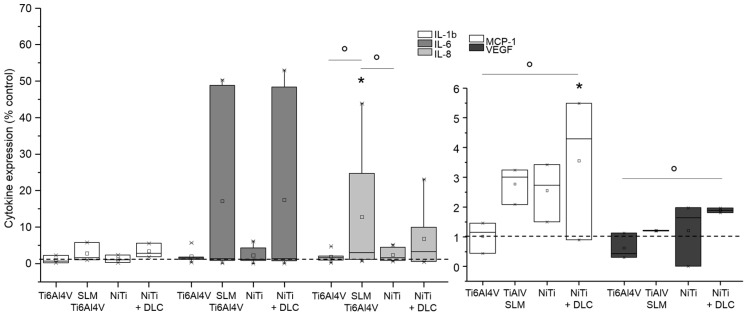
Cytokine level of IL-1b, IL-6, IL-8, MCP-1, and VEGF in supernatants of human osteoblasts (*n* ≥ 2) after 96 h of cultivation on several test pellets (forged Ti6Al4V, Ti6Al4V SLM, NiTi, and NiTi + DLC). Data were normalized to the growth control (=100%, dotted line). Boxes denote interquartile ranges, horizontal lines within the boxes denote medians, and whiskers denote minimum and maximum values. For statistical analysis, ANOVA was conducted. A *p*-value < 0.05 was considered statistically significant. Comparison to growth control: * *p* < 0.05. Comparison between several test pellets: ° *p* < 0.05.

In addition, a cytokine multiplex was conducted to verify the inflammatory cytokine secretion on the metallic test samples. Interleukin syntheses were increased for human osteoblasts cultured on SLM Ti6Al4V and NiTi + DLC (Figure 10, left diagram) compared to the control, while forged Ti6Al4V and NiTi did not show relevant interleukin release. Furthermore, syntheses of MCP-1 and VEGF were higher than on control TCPS for all surfaces except forged Ti6Al4V (Figure 10, right-hand diagram).

**Figure 11:**
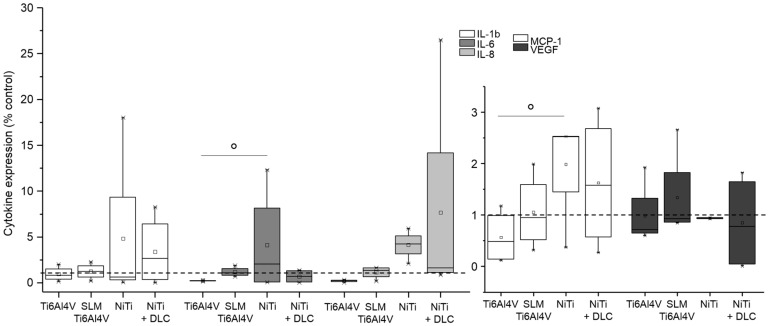
Cytokine level of IL-1b, IL-6, IL-8, MCP-1, and VEGF in supernatants of human fibroblasts (*n* = 4) after 96 h of cultivation on several test pellets (forged Ti6Al4V, Ti6Al4V SLM, NiTi, and NiTi + DLC). Data were normalized to the growth control (=100%, dotted line). Boxes denote interquartile ranges, horizontal lines within the boxes denote medians, and whiskers denote minimum and maximum values. For statistical analysis, ANOVA was conducted. A *p*-value < 0.05 was considered statistically significant. Comparison to growth control: no significances. Comparison between several test pellets: ° *p* < 0.05.

Cultivation of human fibroblasts on the metallic surfaces resulted in slightly increased syntheses of interleukins and MCP-1 mainly on NiTi surfaces compared to the control (Figure 11). In comparison, syntheses were similar to the control or slightly decreased on Ti6Al4V surfaces.

**Figure 12:**
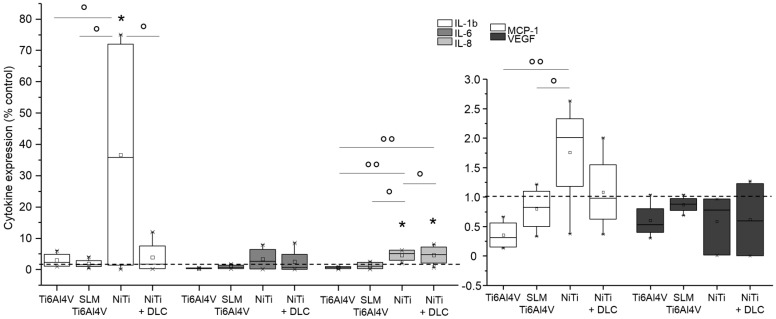
Cytokine levels of IL-1b, IL-6, IL-8, MCP-1, and VEGF in supernatants of human macrophages (*n* ≥ 3) after 96 h of cultivation on several test pellets (forged Ti6Al4V, Ti6Al4V SLM, NiTi, and NiTi + DLC). Data were normalized to the growth control (=100%, dotted line). Boxes denote interquartile ranges, horizontal lines within the boxes denote medians, and whiskers denote minimum and maximum values. For statistical analysis, ANOVA was conducted. A *p*-value < 0.05 was considered statistically significant. Comparison to growth control: * *p* < 0.05. Comparison between several test pellets: ° *p* < 0.05, °° *p* < 0.01.

NiTi surfaces with and without DLC coating resulted in increased interleukin and MCP-1 values compared to controls. This trend was also observed in human macrophages (Figure 12). Cytokine syntheses were not caused by the Ti6Al4V surfaces. Altogether, VEGF syntheses were below the control level for all surfaces.

## 4. Discussion

Four titanium alloys were analyzed with regard to their biocompatibility and inflammatory potential using an osteosarcoma cell line (MG-63) and three human cell types (osteoblasts, fibroblasts, and macrophages). Forged Ti6Al4V, SLM manufactured Ti6Al4V, and NiTi with and without DLC coating samples were used. The elements aluminum and vanadium of the Ti6Al4V alloy may cause neurodegenerative diseases and genotoxicity [19,30]. However, the biological properties of metals like titanium, cobalt-chromium alloys, and stainless steel are well known, since pure titanium and its alloys are associated with the highest biocompatibilities [7,31]. NiTi materials with their high nickel content are also under discussion [31], but their biocompatibility has been proven [15]. When in contact with body fluid (blood, urine, saliva, and serum) or culture medium, all metals tend to corrode, releasing ions into their environments [3,32]. Nevertheless, the intensity of ion dissolution is dependent on the properties of the medium (pH, temperature, and chemical composition) [11,33,34,35] and the dissolution rate and roughness of the metal [34,36]. Although nickel is an essential body element, daily uptake is limited to 600 µg [14]. Hence, in our present in vitro study, all test samples were pre-incubated in culture medium for further cell cultivation in it. Thereby, no negative effect of any of the alloys could be observed compared to the control medium. Supernatants were analyzed after 72 h via Inductively Coupled Plasma–Atomic Emission Spectrometry (ICP-EOS) for testing purposes using an ICP Optical Emission Spectrometers (Varian/Agilent 715-ES, Waldbronn, Germany). Sample digestion and dilution were done with HNO_3_ and with H_2_O_2_ and H_2_O, respectively. Due to technical incidents a high dilution of samples was necessary, so all tested supernatants were under the detectable limit. Cortizo et al. [34] tested ion release from NiTi after 48 h and found that it was also below the detectable limit. Further work revealed little or no influence on cells due to nickel release [37,38]. To avoid a possible ion release, corrosion resistance can be improved by, for example, DLC coating [20,27]. There are opposing statements concerning the influence of uncoated and coated NiTi surfaces. On the one hand, surface coating with DLC is associated with an increased proliferation of osteoblasts and mesenchymal precursors and a higher biocompatibility [22,39,40,41]. Conversely, untreated implant surfaces seemed to be more favorable in other studies [42,43]. In general, DLC coatings are proven to be biocompatible without inducing inflammatory reactions in osteoblasts, fibroblasts, and macrophages and in vivo [27]. In the present in vitro study, the metabolic activity of fibroblasts and macrophages was the lowest with the NiTi sample coated with DLC, but pro-collagen type I synthesis was increased in osteoblasts and fibroblasts. MMP-1 synthesis was significantly increased in osteoblasts, which might explain the low level of collagen 1 synthesis, since MMP-1 is one of the main enzymes degrading collagen 1. In general, MMP-1 synthesis shows similar levels for all configurations, despite a high variability for Ti6Al4V. Nevertheless, results of protein synthesis of CICP and MMP-1 correspond with the results of the appropriate gene expression analysis. Fibroblasts show a nearly reverse behavior, since collagen 1 synthesis is obviously higher than MMP-1 synthesis, although, collagen expression is still slightly decreased on gene level. MMP-1 expression shows extreme variability, but only minor protein synthesis, finally. In gene expression analyses, no inflammatory response was indicated among the samples, but inflammation was slightly detectable in all cell types through the measurement of cytokine and chemokine concentrations in supernatants. Moreover, cell density was the lowest with NiTi coated with DLC for MG-63 cells and macrophages but was unaffected for osteoblasts and fibroblasts. Hence, DLC coating on NiTi seems to be biocompatible, inducing collagen production, but with slight inflammatory reactions. Moreover, our results indicate a promising biocompatibility of SLM manufactured Ti6Al4V and uncoated NiTi since they revealed the highest values of metabolic activity in all tested human cell types and only minor inflammatory reactions, but without an obvious influence on matrix production. Good biocompatibility of NiTi with MG-63 cells [42], osteoblasts [34,43,44,45,46,47], and fibroblasts [33,43,47,48] as well as hMSCs [49] has been proven previously. Furthermore, coated Ti6AlV4 and titanium nitride (TiN)-coated Ti were approved as suitable surfaces by Fleischmann et al. [50]. Fage et al. reported that titanium material can activate macrophages by secreting inflammatory cytokines [12]. NiTi seems to show good biocompatibility in vivo [51,52,53]; however, a slight cytotoxicity of NiTi material to fibroblasts was observed in vitro [54,55]. In general, biocompatibility might depend on the nickel content in the alloy since Bogdanski et al. recommended a content up to about 50% [56] and biocompatibility might result from a TiO_2_ layer created on the material surface similar to titanium [35,57]. Several studies have indicated that the biocompatibility of NiTi is equal to that of pure titanium [32,35,41,53,58,59]. Rocher et al. found that Ti6Al4V and NiTi were the most cytocompatible materials and were equal to cp-Ti [58]. In the end, the cellular response to materials depends on the specific cell types, materials processing, and testing conditions [10,32,60]. Furthermore, surface roughness, topography, and chemistry are crucial factors for cell adhesion, proliferation, and differentiation [3,8,14,61,62,63,64,65]. In our present in vitro study, the Ti6Al4V and NiTi alloys also differed strongly in roughness values. Interestingly, metabolic activity was the highest on the SLM Ti6Al4V samples followed by uncoated NiTi. Although the roughness value Rz varied up to 70 µm, matrix production and inflammatory reactions seemed to be unaffected by surface roughness. Nevertheless, a cell-specific effect on materials with varying surface roughness is well known, as shown in the following. However, adhesion of MG-63 cells is not affected by surface roughness [42]. In contrast, inhibition of MG-63 proliferation and increased osteoblastic differentiation are demonstrated with higher titanium roughness [66]. However, osteoblasts may prefer rougher surfaces [67], but this has not been fully confirmed by other studies [10,21,68]. Kapanen et al. demonstrated the highest cell detachment on rough NiTi and smooth titanium alloyed samples [69]. Moreover, the cell response of osteoblasts to surface roughness depends on their state of maturation [70]. In contrast, a preference for smooth surfaces is clearly displayed by fibroblasts [60,67,68], whereas activation of monocytes/macrophages is caused by rough surfaces [71,72]. Since surface properties of materials are a main factor influencing cell behavior, a variability of results might be based on the use of primary cells. Compared to cell lines, which are cultivated for years, primary cells react in a more specific and sensitive manner. While every single donor can cause variations, even single cells should be taken into account. Further tests could be done analyzing impact of gender or age of the several donors in detail. For example, a high metabolic activity in WST-1 test can be the result of many cells with low or similar activity or of fewer cells with a much higher metabolic activity.

Furthermore, sample specific differences (surface irregularities, residues from manufacturing or cleaning) might influence variability, since a broad range of different manufacturers and processes were deployed.

## 5. Conclusions

In the present in vitro study, the osteoblastic cell line MG-63 as well as human osteoblasts, fibroblasts, and macrophages were cultured on four titanium alloys (forged Ti6Al4V, Ti6Al4V SLM, NiTi, and DLC-coated NiTi) to verify cell-specific biocompatibility and inflammatory potential. Thereby, Ti6Al4V SLM and NiTi revealed the best results regarding metabolic activity. DLC-coated NiTi appeared to be a suitable surface for cell cultivation, with good results for osteoblasts and macrophages. Uncoated NiTi resulted in high collagen production in both mesenchymal cell types. In contrast, forged Ti6Al4V caused an increase of MMP-1 production in osteoblasts and fibroblasts on gene and protein levels. The materials used caused a cell- and surface-dependent inflammatory response. In general, no distinct cell-specific response could be observed regarding the surface material, coating, or roughness. In summary, all of the tested titanium alloys seem to be biologically appropriate for application as orthopedic implants.

## Figures and Tables

**Table 1:** Dimensions as well as values of roughness of the test samples.

Test Pellet	Dimensions (mm^2^)	Rz (µm) Mean ± SD	Ra (µm) Mean ± SD
Ti6Al4V	10 × 2	15.71 ± 1.27	2.38 ± 0.15
Ti6Al4V SLM	10 × 2	68.82 ± 10.59	13.53 ± 2.55
NiTi	10 × 1	1.12 ± 0.26	0.15 ± 0.03
NiTi + DLC	10 × 1	1.13 ± 0.32	0.15 ± 0.02

**Table 2:** Characteristics of the human cell donors used for cell experiments.

Human Cells	Donors	Gender	Average Age
Osteoblasts	*n* = 8	4 ♂/4 ♀	71.88 ± 7.88
Fibroblasts	*n* = 4	− ♂/2 ♀ (2 n/a)	40 ± 7.07 (2 n/a)
Macrophages	*n* = 4	n/a	n/a

**Table 3:** Primers and Primer sequences used for gene expression analysis.

Primer	Primer Sequence
β-Actin	Forward primer: 5′-CTTCCTGGGCATGGAGTC-3′
	Reverse primer: 5′-AGCACTGTGTTGGCGTACAG-3′
Collagen I	Forward primer: 5′-ACGAAGACATCCCACCAATC-3′
	Reverse primer 5′-AGATCACGTCATCGCACAAC-3′
HPRT	Forward primer: 5′-CCCTGGCGTCGTGATTAGTG-3′
	Reverse primer: 5′-TCGAGCAAGACGTTCAGTCC-3′
IL-6	Forward primer: 5′-TGGATTCAATGAGGAGACTTGCC-3′
	Reverse primer: 5′-CTGGCATTTGTGGTTGGGTC-3′
IL-8	Forward primer: 5′-TCTGTGTGAAGGTGCAGTTTTG-3′
	Reverse primer: 5′-ATTTCTGTGTTGGCGCAGTG-3′
MCP-1	Forward primer: 5′-CCGAGAGGCTGAGACTAACC-3′
	Reverse primer: 5′-GGCATTGATTGCATCTGGCTG-3′
MMP-1	Forward primer: 5′-AGAGCAGATGTGGACCATGC-3′
	Reverse primer 5′-TCCCGATGATCTCCCCTGAC-3′
TNF-α	Forward primer: 5′-GTTGTAGCAAACCCTCAAGCTG-3′
	Reverse primer: 5′-GAGGTACAGGCCCTCTGATG-3′

IL: interleukin; MCP-1: monocyte chemoattractant protein-1; MMP-1: matrix metalloproteinase-1; TNF-α: tumor necrosis factor-α.
